# Spatial and socio-demographic predictors of time-to-immunization in a rural area in Kenya: Is equity attainable?

**DOI:** 10.1016/j.vaccine.2010.06.011

**Published:** 2010-08-09

**Authors:** Jennifer C. Moïsi, Jonathan Kabuka, Dorah Mitingi, Orin S. Levine, J. Anthony G. Scott

**Affiliations:** a Department of International Health, Johns Hopkins Bloomberg School of Public Health, Baltimore, MD, USA; b KEMRI/Wellcome Trust Research Programme, Kilifi, Kenya; c Nuffield Department of Clinical Medicine, John Radcliffe Hospital, Oxford, UK

**Keywords:** Immunization coverage, Health inequities, Spatial analysis

## Abstract

We conducted a vaccine coverage survey in Kilifi District, Kenya in order to identify predictors of childhood immunization. We calculated travel time to vaccine clinics and examined its relationship to immunization coverage and timeliness among the 2169 enrolled children (median age: 12.5 months). 86% had vaccine cards available, >95% had received three doses of DTP-HepB-Hib and polio vaccines and 88% of measles. Travel time did not affect vaccination coverage or timeliness. The Kenyan EPI reaches nearly all children in Kilifi and delays in vaccination are few, suggesting that vaccines will have maximal impact on child morbidity and mortality.

## 1 Introduction

The 1980s saw tremendous progress towards universal childhood immunization, as many developing countries received foreign aid and technical support from WHO and UNICEF to build and sustain national immunization programs. By 1990, coverage with three doses of Diphteria–Tetanus–Pertussis vaccine (DTP3) was said to have attained 79% globally, though sub-Saharan Africa and southern Asia lagged behind other regions, with only 52% and 68% coverage. Limited improvements in coverage have been achieved since 1990 [1], but new efforts are underway to establish universal immunization. As part of the polio eradication initiative, many countries conduct national and sub-national “catch-up” campaigns to vaccinate all children, and the GAVI Alliance has supplied funding for strengthening routine immunization services since 2000.

Despite these efforts, available data show that inequalities in immunization coverage persist across the developing world [2]. At the country level, stark variations in coverage exist among socio-economic groups, and in some cases between sexes [3,4]. Further, expansions in coverage do not always produce improvements in equity [5]. Supplementary immunization activities may serve to reduce these disparities, but they are limited to polio and measles vaccines and therefore have no benefit for other target diseases. At the local level, studies have shown increases in coverage with socio-economic status, higher coverage in non-migrant than in migrant populations, and delayed administration of vaccines in the rainy season, in remote areas, and in larger families [6–10]. Though a large body of literature has demonstrated that the likelihood of seeking curative care decreases with increasing distance to health facilities [11–14], analogous data on immunization are limited and inconsistent [6,9,15–18]. Children living far from clinics may have the highest mortality risk [10,19,20], supporting the need to investigate whether they have equitable access to immunization services.

With support from GAVI, Kenya plans to introduce pneumococcal conjugate vaccine (PCV) into its routine immunization schedule in 2010. Vaccine coverage surveys in Kilifi District, Kenya before and after the introduction of Hib vaccine showed that 88–100% of children in this area were immunized with three doses of DTP or DTP-Hepatitis B-Hib (pentavalent) vaccine, but that many received their vaccines late [9] mirroring findings from DHS surveys conducted in several developing countries [2]. For diseases with high incidence in the first few months of life such as *Haemophilus influenzae* type b or *Streptococcus pneumoniae* infections, delays in immunization may diminish the impact of vaccine even if coverage at age 12 months is high. In this context, we sought to identify predictors of the timing of immunization among infants in Kilifi District, with a focus on the effect of spatial factors such as distance to vaccine clinics.

## 2 Methods

### 2.1 Study site

This study was conducted in Kilifi District, a largely rural area situated on the Indian Ocean coast of Kenya. In 2000, the Kenya Medical Research Institute (KEMRI)/Wellcome Trust Research Programme established an Epidemiologic and Demographic Surveillance System (Epi-DSS) to monitor vital events and migrations in a 900 km^2^ area around the hospital covering over 220,000 people. Approximately three census rounds have been completed each year since the initial population enumeration. A survey of health facilities conducted in September 2006 identified 47 public, private, or NGO-run immunization clinics serving Kilifi District, 16 of which are located within the Epi-DSS area. The Kenyan EPI recommends that children receive Bacillus Calmette-Guerin (BCG) and Oral Polio Vaccine (OPV) at birth; three doses of pentavalent vaccine and OPV at 6, 10 and 14 weeks of age; and measles vaccine at 9 months of age. Immunizations are recorded on vaccine cards or booklets obtained from the clinics.

### 2.2 Immunization study subjects

All children residing in the Epi-DSS area were eligible for enrollment within 1 month of their first birthday. Using the Epi-DSS population register, we selected a 30% simple random sample of eligible children each month from January to October 2007 and 20% in November and December 2007. We aimed to enroll at least 1904 children in order to have 90% power to detect a five percentage-point difference in coverage with three doses of pentavalent vaccine between areas close to immunization clinics (assumed to have 90% coverage) and areas far from clinics, at a significance level of 0.05.

### 2.3 Immunization data collection

Field workers visited the homes of all children selected for study participation. After obtaining informed consent from the mother or guardian, they completed a questionnaire listing the date and location each vaccine was received based on the child's vaccination card or on maternal recall (if the card was unavailable). Given the target age at enrollment, very few post-infantile vaccinations were recorded. When the first home visit was unsuccessful, up to two follow-up visits were conducted unless (1) the mother/guardian refused to participate; (2) the mother/guardian had migrated to an area outside the Epi-DSS, or to an unknown destination within the area; (3) no child meeting the study inclusion criteria resided at the homestead due to an Epi-DSS register error. Mothers who had migrated within the Epi-DSS area were sought in their new residence.

### 2.4 Mapping and travel time estimation

The Epi-DSS area has been thoroughly mapped using Magellan (Magellan Navigation Inc., Santa Clara, CA) and e-Trex (Garmin Ltd., Olathe, KS) Geographic Positioning Systems (GPS) technology, including administrative location boundaries, homestead coordinates, footpaths, roads, and matatu (local bus) routes with associated transport speeds. All geographic data were imported via Datasend, Map Source, or DNRGarmin software into ArcGIS 9.2 (ESRI, Redlands, CA) for mapping and analysis.

Travel time to vaccine clinics was calculated using an ArcGIS cost-distance algorithm. The details of this method have been described elsewhere [21]. We constructed an impedance raster (a grid in which each cell is assigned a friction or inverse speed value) to define the speed of travel through each 100-m × 100-m area of Kilifi District, assuming speeds of 5 km/h on roads and footpaths and 2.5 km/h off-road for pedestrian travel, and matatu speeds on matatu routes for vehicular travel. The algorithm uses the raster to calculate a catchment area for each health facility and travel time to this facility from all homesteads in its catchment area.

### 2.5 Vaccine coverage analysis

We calculated the proportion of children receiving a given dose of vaccine (“vaccine coverage”) among subjects with and without vaccine cards, by sex, administrative location, ethnic group (individual categories for groups with at least 25 survey participants, “other” category combining groups with <25 participants), maternal education (proportion of women 15–49 years old with any education in a given sublocation: group 1, <0.5; group 2, 0.5 to <0.6; group 3, 0.6 to <0.7; and group 4, ≥0.7),^1^ migrant status (migrant: migration from outside the Epi-DSS area between 2000 and 2006), and month of birth, and compared coverage across strata using chi-square tests. For children with vaccine cards, we obtained coverage at specific time points and median and inter-quartile ranges for age at vaccination. We constructed inverse Kaplan–Meier survival curves for immunization with one, two and three doses of pentavalent vaccine and compared time-to-immunization across strata using log-rank tests. We built multivariable Cox proportional hazards models to investigate the effects of travel time to vaccine clinics, sex, ethnic group, maternal education, migration and season (rainy: April–June and October–November) on time-to-immunization with any dose of pentavalent vaccine, with each child contributing survival time from 14 days of age for dose one and from the date of the previous dose for doses two and three. Children with missing dates of vaccination were excluded from individual analyses as appropriate. We used a spatial bootstrap method with 100 repetitions to account for the intra-subject correlation induced by repeat observations from individual children and the inter-subject correlation engendered by spatial clustering of immunization events. In each repetition, we randomly selected 40 sublocations (with replacement) and estimated the proportional hazards model on all data from the selected sublocations. Variables without statistically significant effects (at the 0.05 level) based on Wald tests were dropped from the multivariable models. Complementary log–log graphs and Wald tests for time-varying covariates were used to assess the validity of the proportional-hazards assumption. All analyses were conducted in Stata 9.2 (StataCorp, College Station, TX).

## 3 Results

### 3.1 Enrollment

We randomly selected 2504 eligible subjects from the population register. Of these, 1804 were enrolled on the first home visit and an additional 271 (of 509), 82 (of 180) and 12 (of 28) were enrolled on a second, third and fourth visit, for an overall enrollment rate of 86.6% (2169/2504). Reasons for non-enrollment included refusal to participate (23, 6.9%), loss to follow-up after three or more unsuccessful visits (77, 23%), out-migration to an unknown location (48, 14.3%), out-migration outside the Epi-DSS area (136, 40.6%), database error (e.g. mapping error, age error: 47, 14%), and fieldwork error (4, 1.2%). Enrollment attained 95.4% when out-migrants and database errors were excluded. Monthly enrollment ranged from 79% to 93.7%, with 155–303 subjects visited each month (83 in December 2007).

Survey respondents for the 2169 enrolled children included 1859 mothers, 131 fathers and 179 other relatives. Vaccine cards were available for 1870 subjects (86.2%). Enrolled children were born between March 29th, 2005 and December 31st, 2006. Median age at enrollment was 12.5 months (IQR: 12.0–13.1) and did not vary over the course of the study (11.8–13.3 months). Children less than 11 months of age at enrollment were excluded from further analyses (*N* = 41).

Vaccine card retention varied by location, ranging from 76.6% to 96.4% (*p* = 0.01). Children without cards (*N* = 296) were more likely to be girls than those with cards (*N* = 1832) (55% vs. 47%, *p* = 0.01), but were not significantly different with regard to ethnic group or maternal education.

### 3.2 Coverage in children with and without vaccine cards

Coverage in children with cards was high, attaining 98.9% for BCG, 95.7% for three doses of pentavalent vaccine, 95.6% for three doses of OPV and 89.7% for measles vaccine. Three-quarters of vaccinated children received their vaccines within 1 month (30 days) of the recommended age for all but the third doses of pentavalent and OPV, for which the 75th percentile was reached 44 and 38 days late, respectively (Table 1).

For all vaccines except the birth dose of OPV, coverage was three to seven percentage points higher for children with vaccine cards than for children without vaccine cards, and the differences in coverage were statistically significant (*p* < 0.001) (Table 2). Only OPV0 coverage was higher by maternal recall than by card (86.2% vs. 51.1%, *p* < 0.001).

### 3.3 Coverage by sex, location, ethnic group, maternal education, migrant status and month of birth

**Fig. 1:**
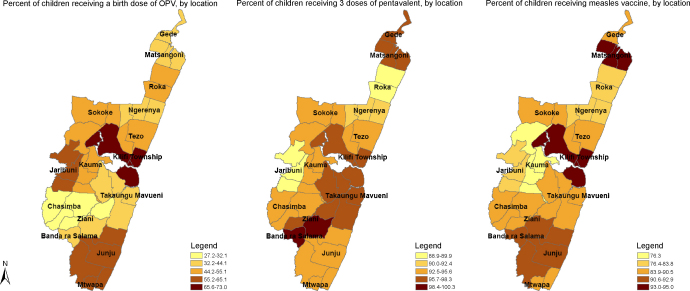
Immunization coverage for OPV0, Penta3, and measles vaccines at time of survey, by location.

In children with vaccine cards, coverage varied by geographic location for OPV0 (27.2% in Ziani to 73% in Kilifi Township, *p* < 0.001), Penta3 (88.9% in Jaribuni to 100% in Banda ra Salama, *p* = 0.02), OPV3 (88.1% in Roka to 98.8% in Banda ra Salama, *p* = 0.01) and measles vaccine (76.3% in Kauma to 95% in Kilifi Township, *p* < 0.001); coverage was similar across locations for all other vaccines (Fig. 1). Coverage varied by month of birth for BCG, OPV0 and OPV1, ranging from 96.6% to 100%, 35.5% to 58.8%, and 96.4% to 100% respectively, with no seasonal patterns. Coverage by sex, ethnic group, maternal education, and migrant status for each of the vaccines is shown in Table 3. With the exception of OPV0, there were limited variations in coverage across categories for each of these attributes.

### 3.4 Time-to-immunization

**Fig. 2:**
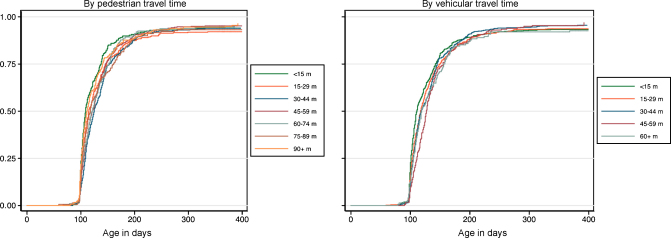
Time-to-immunization with three doses of pentavalent vaccine, by pedestrian and vehicular travel time to vaccine clinics.

Pedestrian and vehicular travel times to vaccine clinics ranged from 0 to 170 min (median: 47 min, inter-quartile range 27–73) and 0 to 132 min (median: 27 min, inter-quartile range 14–40), respectively. Log-rank tests showed differences in time-to-immunization with two or three doses of pentavalent vaccine across pedestrian travel time strata (*p* = 0.02), but no clear trends with either pedestrian or vehicular travel time (Fig. 2).

Travel time was not associated with time-to-immunization with pentavalent vaccine in bivariate or multivariable proportional hazards models (HR = 1.00 for pedestrian and HR = 1.01 for vehicular travel time). In bivariate models, children in the most educated areas had higher immunization rates than those in less educated areas (HR[group 4 vs. groups 1–3] = 1.22, 95% CI 1.17–1.28) and migrant children had slightly higher rates than non-migrants (HR = 1.04, 95% CI 1.01–1.07), but these effects disappeared after adjusting for travel time. The only significant predictor of immunization rates in the final model was season, with lower rates observed in the rains than in the dry season (HR = 0.86, 95% CI: 0.81–0.92).

## 4 Discussion

This large-scale survey of young children in Kilifi District, Kenya showed very high immunization coverage for all recommended vaccines, with 98.9%, 95.7%, 95.6% and 89.7% of subjects with vaccine cards receiving BCG, three doses of pentavalent, three doses of OPV, and measles vaccines by the age of 1 year, respectively. Only 14% of enrolled subjects did not have vaccine cards available for examination. In this group, reported coverage was three to seven percentage points lower for all doses of vaccine (except OPV0), but remained >90% for BCG, DTP-HepB-Hib3, OPV3 and >80% for measles. The wide discrepancy between maternal reporting and card data for OPV0 coverage is specific to this vaccine, and may reflect poor recall for the period immediately after delivery. The reliability of mothers’ histories was previously evaluated in this setting among 18 children enrolled in a small immunization coverage survey, showing that 100% of mothers correctly recalled the first dose of DTP, 94% the second dose and 88% the third dose [9]. Evidence from other regions is conflicting, with some studies suggesting that maternal recall has low accuracy [22–27]. Most of these studies were conducted in industrialized countries and data from Kilifi, Egypt [23] or Sudan [28] may be more relevant for our analysis. Regardless of the reliability of maternal recall, we calculated that even with 0% coverage in children without cards, overall coverage for BCG, Pentavalent-3 (or OPV3) and measles would attain 85%, 82% and 77%, respectively; these values would increase to 92%, 89% and 84% with 50% coverage in children without cards. In addition to recall bias, our results may be subject to survivor bias because we only sampled children who were alive and 6–11 months of age at the time of the last Epi-DSS census. The 2006 birth cohort had an infant mortality ratio of 37 per 1000 live births (unpublished data, Kilifi Epi-DSS): even if none of these children were vaccinated, BCG, pentavalent-3, and measles coverage would only be reduced to 95%, 92% and 86%, respectively. Together, these results strengthen the evidence from earlier, smaller studies conducted from 2002 to 2004 [9], and attest to the success of the Kenyan EPI in reaching a large majority of children in Kilifi. They also concur with data from the 2008 Kenya Demographic and Health Survey (unpublished data, Kenya 2008 DHS) and WHO/UNICEF joint estimates [29] that showed approximately 95% coverage with BCG, 85% with Penta3, and 85–90% with measles vaccine on a national level.

We sought to investigate spatial variations in immunization coverage, and found that these were relatively limited in the study area. For Penta3, OPV3 and measles, we observed disparities across administrative locations, with 89–100% coverage for Penta3, 88–99% for OPV3 and 80–92% for measles. Time-to-immunization varied by location as well: children in Kilifi Township received each dose of pentavalent vaccine earlier than their peers in rural areas. However, the hypothesis that improved physical access to vaccine clinics increases the timeliness of immunization was not substantiated by our data. This finding may stem from a number of factors. First, travel time to vaccine clinics varied little within the Epi-DSS. Maximum pedestrian and vehicular travel times to vaccine clinics were less than 3 h and less than 2.5 h, respectively, with 75% of children residing less than 72 min on foot and less than 42 min by vehicle from a clinic. In this context, traveling to clinics may not impose a significant burden on families or hamper timely immunization. Second, we were unable to account for several factors that may confound the association between time-to-immunization and physical access to care. We employed sublocation-level maternal education as a proxy for socio-economic status and were therefore unable to reflect inequalities in socio-economic status within sublocations, which may be associated both with distance to clinics and timing of immunization. Further, we were unable to account for family size or birth intervals in our model. Parity and birth intervals may affect time-to-immunization and are likely to vary with distance to clinics; they may therefore be important confounders as well [9,30].

We have previously shown that travel time is a barrier to hospital admission in the Kilifi Epi-DSS (J Moïsi, submitted). Assuming no residual confounding, the absence of a relationship between timeliness of vaccination and distance to clinics in this analysis suggests that programmatic differences between immunization and hospital service delivery play an important role in service utilization. Programmatic factors contributing to high immunization coverage may include the decentralized provision of immunization services, the perceived high quality of these services, or the focus on proactive outreach efforts via Supplementary Immunization Activities (SIAs) and mobile clinic team activities. Measles and polio SIAs were conducted in Kilifi District in the second half of 2006, but should have no effect on pentavalent vaccine coverage since the vaccine was not delivered through this mechanism. Outreach via mobile teams was donor-funded, localized, and sporadic during the study period yet may have contributed to high coverage as well.

While no variations in time-to-immunization were seen with travel time to vaccine clinics, other key predictors of immunization rates were identified in this study. At a given age, children were 14% less likely to be immunized with pentavalent vaccine during the rains than during the dry season: the rainy season coincides with the harvest and impedes travel, even for short distances. However, coverage with three doses of pentavalent and OPV vaccines at age 12 months did not vary by month of birth, suggesting that delays in receiving individual doses do not correlate with a long-term decrease in the likelihood of vaccination. Secondly, residing in an area with high levels of maternal education or belonging to a migrant family was associated with an increase in immunization rates in bivariate analyses. These effects disappeared in multivariable analyses, reflecting possible confounding by travel time to vaccine clinics. Overall, however, the effect of maternal education produced higher coverage with three doses of pentavalent vaccine at age 12 months in the most educated areas compared to the less educated ones. This result is consistent with 2008 Kenya DHS data showing substantially higher coverage for all vaccines in children with educated mothers compared to those with uneducated mothers (unpublished data, Kenya 2008 DHS), and buttresses the notion of a strong relationship between maternal education and child health.

Geographic access to care in the Kilfi Epi-DSS is comparable to most other regions of Kenya [31] and immunization coverage is similarly high based on data from the most recent Demographic and Health Survey and WHO/UNICEF joint coverage estimates. It is therefore likely that the vast majority of Kenyan children enjoy as equitable and timely access to immunization as do residents of our study area. In this context, the introduction of a new, effective vaccine against pneumococcal disease is likely to reach all children at an early age and lead to substantial improvements in child health.

## Figures and Tables

**Table 1:** Cumulative vaccine coverage among children with vaccination cards and age at vaccination for immunized children.

	Age											Median age (days)	IQR (days)
	2 weeks (%)	4 weeks (%)	6 weeks (%)	10 weeks (%)	14 weeks (%)	18 weeks (%)	22 weeks (%)	9 months (%)	10 months (%)	11 months (%)	12 months (%)		
BCG	31.8	56.4	69.8	86.9	93.7	96.5	97.6	98.7	98.8	98.9	98.9	22	10–44
Pentavalent 1	–	–	8.8	80.1	94.6	97.6	98.6	99.4	99.5	99.6	99.7	46	42–62
Pentavalent 2	–	–	–	5.9	70.1	88.2	93.7	98.1	98.4	98.5	98.8	80	72–101
Pentavalent 3	–	–	–	–	3.4	57.9	78.3	94.3	94.9	95.4	95.5	116	103–142
OPV0	34.7	46.0	48.7	49.8	49.9	49.9	49.9	50.1	50.1	50.1	50.1	9	3–15
OPV1	–	–	10.4	83.7	96.3	98.2	98.8	99.5	99.6	99.6	99.7	46	42–68
OPV2	-	–	–	6.7	73.4	91.1	94.8	98.3	98.5	98.5	98.8	79	72–98
OPV3	–	–	–	–	4.2	61.2	82.0	94.5	94.9	95.2	95.4	114	102–137
Measles	–	–	–	–	–	–	–	35.4	72.8	83.7	88.1	277	264–296

**Table 2:** Percentage of children with and without immunization cards who had received each of the EPI vaccines^a^.

Vaccine	Vaccine coverage at 12 m among those with a card (%)	Vaccine coverage at 12 m among those without a card (%)
BCG	99.0	94.3
Pentavalent 1	99.7	95.3
Pentavalent 2	98.8	93.6
Pentavalent 3	95.7	91.2
OPV0	51.1	86.2
OPV1	99.7	94.9
OPV2	98.9	94.6
OPV3	95.6	92.6
Measles	89.7	82.8

a If vaccination status was unknown, we assumed vaccine was not received.

**Table 3:** Percentage of children with vaccination cards who had received each of the EPI vaccines, by sex, ethnic group, maternal education and migrant status.

	BCG	Penta1	Penta2	Penta3	OPV0	OPV1	OPV2	OPV3	Measles
*Sex*									
Female	99.2	99.8	99.0	95.3	50.9	99.8	99.0	95.7	88.8
Male	98.8	99.6	98.7	95.6	51.3	99.6	98.7	95.5	90.4
*p*-value*	0.38	0.51	0.57	0.81	0.88	0.51	0.57	0.83	0.25

*Ethnicity*									
Giriama	99.0	99.5	98.9	95.9	52.6	99.7	98.9	95.6	91.3
Chonyi	99.2	100.0	98.8	96.3	42.7	99.8	98.8	95.7	88.1
Kauma	98.5	99.0	98.5	94.3	50.5	99.5	98.5	94.9	83.5
Duruma	100.0	100.0	100.0	96.4	60.7	100.0	100.0	96.4	92.9
Jibana	98.3	100.0	100.0	93.1	65.5	100.0	100.0	100.0	93.1
Other	98.3	100.0	98.3	94.1	77.1	99.2	98.3	94.9	94.1
*p*-value	0.88	0.32	0.95	0.75	**<0.01**	0.88	0.95	0.88	**<0.01**

*Education*									
<0.5	99.1	99.6	98.7	96.1	42.2	100.0	98.7	95.7	85.7
0.5- < 0.6	99.4	99.7	99.0	96.7	47.3	99.6	99.2	96.7	90.4
0.6- < 0.7	98.6	99.5	98.1	92.3	53.5	99.5	97.6	92.3	87.7
≥0.7	98.2	100.0	99.4	98.2	80.4	100.0	99.4	97.6	95.8
*p*-value	0.54	0.81	0.44	**<0.01**	**<0.01**	0.63	0.08	**<0.01**	**<0.01**

*Migration*									
Non-mig	98.9	99.7	98.7	95.8	49.5	99.7	98.8	95.7	89.3
Migrant	99.4	99.7	99.0	94.8	58.3	99.7	98.7	94.8	90.9
*p*-value	0.45	0.98	0.67	0.44	**<0.01**	0.98	0.88	0.50	0.39

Statistically significant findings are indicated in bold.

* *p*-values based on a chi-square tests for proportion.

## Footnotes

1 Education data were not collected in the immunization coverage survey. We therefore used data obtained from all residents of the Epi-DSS during the sixth census round (April–August 2004) to calculate the proportion of women of childbearing age with any schooling in each administrative sublocation and used it to generate a sublocation-level categorical variable (proportion of mothers with any education <0.5; 0.5 to <0.6; 0.6 to <0.7; and ≥0.7), which was then applied to individuals based on their residence.
